# Coagulation and Fibrinolysis Index Profile in Patients with ANCA-Associated Vasculitis

**DOI:** 10.1371/journal.pone.0097843

**Published:** 2014-05-19

**Authors:** Tian-Tian Ma, Yi-Min Huang, Chen Wang, Ming-Hui Zhao, Min Chen

**Affiliations:** 1 Renal Division, Department of Medicine, Peking University First Hospital, Peking University Institute of Nephrology, Beijing, China; 2 Key Laboratory of Renal Disease, Ministry of Health of China, Beijing, China; 3 Key Laboratory of Chronic Kidney Disease Prevention and Treatment (Peking University), Ministry of Education, Beijing, China; 4 Peking-Tsinghua Center for Life Sciences, Beijing, China; Gentofte University Hospital, Denmark

## Abstract

**Background:**

Previous studies observed the high prevalence of venous thromboembolism in patients with antineutrophil cytoplasmic antibody (ANCA)-associated vasculitis (AAV). The current study analyzed the coagulation and fibrinolysis index profile in AAV patients.

**Methods:**

The current study recruited 321 AAV patients in active stage and 78 AAV patients in quiescent stage. Coagulation and fibrinolysis index profiles in these AAV patients were analysed, and their associations with various clinical and pathological parameters were further investigated.

**Results:**

The circulating levels of D-dimer, fibrin degradation products and platelet count were significantly higher in AAV patients in active stage compared with those in remission [0.8 (0.4, 1.5) mg/L vs. 0.28 (0.2, 0.55) mg/L, P<0.05; 5.6 (5.0, 10.0) mg/L vs. 1.9 (1.2, 2.8) mg/L, P<0.05; 269±127×10^9^/L vs. 227±80×10^9^/L, P<0.05, respectively]. Among the 321 AAV patients in active stage, compared with patients with normal levels of D-dimer, patients with elevated D-dimer levels had significantly higher levels of initial serum creatinine, erythrocyte sedimentation rate, C reactive protein and the Birmingham Vasculitis Activity Scores (P = 0.014, P<0.001, P<0.001, P = 0.002, respectively). Moreover, correlation analysis showed that the levels of D-dimer correlated with erythrocyte sedimentation rate and C reactive protein levels (r = 0.384, P<0.001; r = 0.380, P<0.001, respectively).

**Conclusion:**

Patients with active AAV are in hypercoagulable states, and circulating levels of D-dimer are associated with disease activity of AAV.

## Introduction

Antineutrophil cytoplasmic antibody (ANCA)-associated vasculitis (AAV) is a group of systemic vasculitis associated with ANCA specific for myeloperosidase (MPO) or proteinase-3 (PR3). AAV includes granulomatosis with polyangiitis (GPA), microscopic polyangiitis (MPA), and eosinophilic granulomatosis with polyangiitis (EGPA) [1]. The high risk of acute venous thrombosis in AAV was initially recognized in the pediatric population [2] and confirmed in a large randomized trial conducted by the Wegener's Granulomatosis Etanercept Trial Research Group [3]. In a retrospective study, Stassen et al. found the overall incidence of venous thromboembolism (VTE) in patients with AAV was 1.8/100 person-years, and increased to 6.7/100 person-years in periods with active AAV [4]. A higher prevalence of venous thrombosis has been observed in patients with AAV compared with healthy population of the same age. Merkel et al. prospectively investigated VTE in patients with GPA, and reported an incidence of 7.0/100 person-years of VTE in GPA patients [5]. However, the coagulation and fibrinolysis index profile in patients with AAV was not clear yet. In this retrospective study, we analyzed the coagulation and fibrinolysis index profile in AAV patients in both active and quiescent phases, and their associations with various clinical and pathological parameters were further investigated.

## Patients and Methods

### Patients

The current study retrospectively recruited 321 consecutive patients with newly onset AAV diagnosed in Renal Division, Peking University First Hospital between July 1998 and November 2011. All these patients met the Chapel Hill Consensus Conference criteria for AAV [1]. Exclusion criteria was defined as follows: (1) patients with negative ANCA; (2) patients with secondary vasculitis, such as drug-induced vasculitis; or with comorbid renal diseases, for instance, anti-glomerular basement membrane disease, lupus nephritis, IgA nephropathy, or diabetic nephropathy; (3) patients with EGPA, since EGPA is increasingly considered a distinct type of AAV with different manifestations and outcomes as compared to GPA and MPA [6]. Disease activity was assessed in accordance with the Birmingham Vasculitis Activity Score (BVAS) [7]. Plasma samples of 78 patients with AAV, who achieved remission after immunosuppressive therapy, were also collected at their regular ambulatory visits. “Remission” was defined as “absence of disease activity attributable to active disease qualified by the need for ongoing stable maintenance immunosuppressive therapy” (complete remission), or “at least 50% reduction of disease activity score and absence of new manifestations” (partial remission), as described previously [8]. The research was in compliance of the Declaration of Helsinki and approved by the ethics committee of the Peking University First Hospital. Written informed consent was obtained from each participant. As for the children, written informed consents were obtained from their guardians on behalf of them.

### Detection of serum ANCA

ANCA tests were performed by both indirect immunofluorescence assay and antigen-specific enzyme-linked immunosorbent assay. Both the tests for ANCA were performed according to the manufacturer (Euroimmun, Lübeck, Germany). In indirect immunofluorescence assay, cytoplamic ANCA (cANCA) and perinuclear ANCA (pANCA) were distinguished. In antigen-specific enzyme-linked immunosorbent assay, ANCA directed to proteinase 3 (PR3) and myeloperoxidase (MPO) were tested. For those patients with diverse results from these two assays, we used the results of antigen-specific enzyme-linked immunosorbent assay.

### Thromboembolic events and coagulation and fibrinolysis index of the patients

We recorded thromboembolic events and collected the coagulation and fibrinolysis index profile of these patients. The thromboembolic events were recorded according to vascular ultrasound and computed tomography. Since this was a retrospective analysis, the individual method employed was based solely on the treating physician's choice. The coagulation and fibrinolysis index, including plasma prothrombin time (PT) (Nycotest PT, Axis-Shield Poc As, Oslo, Norway, the normal range of PT is between 9.8 and 12.4 sec), activated partial thromboplastin time (APTT) (Actin FSL Siemens Healthcare Diagnostics, Marburg, Germany, the normal range of APTT is between 26.9 and 37.6 sec), D-dimer (Tina-quant D-dimer, Roche Diagnostics, Mannheim, Germany, the normal range of D-dimer is between 0.1–0.5mg/L) and fibrin degradation products (FDP) (SpliPrest, Diagnostica Stago, the normal range of FDP is between 0 and 5 mg/L), were measure by the Central Laboratory Department of our hospital. The D-dimer was in Fibrinogen units. The International Sensitivity Index (ISI) value for the PT assay ranged from 0.96 to 0.99. During the fibrinolysis, dissolution of crosslinked fibrin leads to formation of specific degradation products, including D-dimer [9]. A normal D-dimer value excludes the diagnosis of venous thrombosis, while an elevated value supports it [10].

## Results

### Demographic and general data

Of the 321 patients with AAV in active stage, 155 were male and 166 were female, with an age of 63±14.6 (range14–89) years at diagnosis, including three children, with an age of 14, 14 and 16 respectively. The level of initial serum creatinine was 439±345.1 (range 55–1759) µmol/L. The level of urinary protein excretion was 2.0±1.66 (range 0.04–13.09) g/24 hr. The level of erythrocyte sedimentation rate (ESR) was 68.2±39.6 mm/1 hr. The level of BVAS was 19.9±6.8. The general data of the patients with AAV were listed in Table 1. For the 78 patients with AAV in remission stage, the levels of BVAS were 0 and 1 in 76 and 2 patients, respectively.

**Table 1:** General data of patients with AAV in acute phase.

	Values
Total number of patients	321
Gender (male/female)	155/166
Age at diagnosis of diaease (years)	63±14.6
MPO-ANCA/PR3-ANCA	292/29
Initial Scr ( µmol/L)	
Mean±s.d.	439±345.1
Range	55–1759
Urinary protein (g/24 hr)	
Mean±s.d.	2.0±1.66
Range	0.04–13.09
ESR (mm/1 hr)	68.2±39.6
BVAS	19.9±6.8
Renal involvement	303(94.4%)
Skin rash	60(18.7%)
Arthralgia	65(20.2%)
Muscle pain	74(23.1%)
Pulmonary involvement	222(69.2%)
ENT involvement	137(42.7%)
Ophthalmic involvement	70(21.8%)
Gastrointestinal involvement	40(12.5%)
Nervous system involvement	68(21.1%)

[Abbreviations]: AAV, anti-neutrophil cytoplasmic antibody-associated vasculitis; ANCA, antineutrophil cytoplasmic antibodies; BVAS, Birmingham Vasculitis Activity Scores; ENT, ear, nose, and throat; ESR, erythrocyte sedimentation rate; MPO, myeloperoxidase antibodies; PR3, proteinase 3 antibodies; s.d., standard deviation; Scr, serum creatinine.

### Venous thromboembolism (VTE)

Among the 321 patients with AAV, there were totally 13 patients who developed VTEs during the active stage of the disease. Among these 13 patients, 12 patients were classified as MPA and the other one was classified as GPA. Nine out of the 13 patients had VTE on the lower extremities, 1/13 patient had pulmonary embolism, 2/13 patients had thrombosis in the renal vein, and the other 1/13 patient had thrombosis in the jugular vein. However, none of the 78 patients with AAV in remission stage developed VTEs. No significant difference of the occurrence of VTEs was found between patients with PR3-ANCA and MPO-ANCA.

### Coagulation and fibrinolysis index profiles in AAV patients in active stage and remission

Among the 321 AAV patients in active stage, the level of PT was 11.8±1.7 (range 8.3–26.7) sec. The level of APTT was 31.8±12.5 (range 20.4–214.7) sec. The level of FDP was 5.6 (5.0, 10.0) mg/L. The level of D-dimer was 0.8 (0.4, 1.5) mg/L. The level of platelet count was 269±127 (range 38–739) ×10^9^/L.

The level of D-dimer, which is a useful aid in the diagnosis of thromboembolism, was significantly higher in AAV patients in active stage compared with that in remission [0.8 (0.4, 1.5) mg/L vs. 0.28 (0.2, 0.55) mg/L, P<0.05]. The levels of FDP and platelet count were also significantly higher in AAV patients in active disease compared with AAV patients in remission [5.6 (5.0, 10.0) mg/L vs. 1.9 (1.2, 2.8) mg/L, P<0.05; 269±127 ×10^9^/L vs. 227±80×10^9^/L, P<0.05, respectively].

### Associations between D-dimer levels and clinical features

Among 321 AAV patients in active stage, there were 221 patients whose circulating D-dimer levels were higher than the normal range (≥0.5 mg/L), and the circulating D-dimer levels among the other 100 patients were in the normal range (<0.5 mg/L). Compared with patients without elevated D-dimer levels, those with elevated D-dimer levels had significantly higher levels of initial serum creatinine (P = 0.014). The levels of ESR, C reactive protein (CRP) and BVAS, which are useful markers reflecting disease activity of AAV, were significantly higher in patients with elevated D-dimer levels than those in patients with normal D-dimer levels (P<0.001, P<0.001, P = 0.002, respectively). Compared with patients with normal D-dimer levels, patients with elevated D-dimer levels had significantly higher levels of white blood cell count and platelet count, and significantly lower level of hemoglobulin in the peripheral blood cell count test (P = 0.01, P = 0.001, P<0.001, respectively) (Table 2). Moreover, correlation analysis showed that the levels of D-dimer correlated with ESR and CRP levels (r = 0.384, P<0.001; r = 0.380, P<0.001, respectively). No significant difference of the coagulation and fibrinolysis index was found between patients with PR3-ANCA and MPO-ANCA.

**Table 2:** Comparison of clinical and laboratory features of AAV patients with and without elevated levels of D-dimer in active stage.

	D-dimer≥0.5 mg/L (n = 221)	D-dimer<0.5 mg/L (n = 100)	P
Initial Scr, µmol/L(median,IQR)	407.0 (182.5/657.0)	252.0 (110.5/583.5)	P = 0.014
ESR,mm/1 h (median,IQR)	75.0 (46.5/110.0)	40.00 (21.8/67.3)	P<0.001
CRP,mg/L (median,IQR)	25.8 (8.0/72.5)	7.54 (2.2/17.1)	P<0.001
Platelet count, ×10^9^/L (median,IQR)	268.0 (186.0/371.0)	217.0 (151.0/296.0)	P = 0.001
BVAS (mean,s.d.)	20.4±6.7	17.8±6.8	P = 0.002
WBC, ×10^9^/L (median,IQR)	9.9 (7.4/12.6)	8.4 (6.0/12.1)	P = 0.010
Hb,g/dL(median,IQR)	8.6 (7.2/10.1)	9.6 (8.2/11.2)	P<0.001

[Abbreviations]: AAV, anti-neutrophil cytoplasmic antibody–associated vasculitis; BVAS, Birmingham Vasculitis Activity Scores; CRP, c-reactive protein; ESR, erythrocyte sedimentation rate; Hb, hemoglobin; IQR, interquartile range; Scr, serum creatinine; WBC, white blood cell.

## Discussion

AAV is a group of autoimmune disorders. In some previous studies, it has been observed that patients with AAV have an increased risk of developing VTEs, especially when AAV is active [4], [5]. The current study investigated the coagulation and fibrinolysis index profile in AAV patients, and their associations with various clinical and pathological parameters.

The current study found the abnormal fibrinolysis index of patients with AAV in the active stage, characterized by elevated levels of circulating FDP and D-dimer, which supported thrombosis formation [9], [10] and hypercoagulable state. There are several lines of the potential mechanism. For example, neutrophil extracellular traps (NETs), a meshwork of DNA fibers comprising histones and antimicrobial proteins, should be one of the important contributors. The level of NETs is increased in AAV patients, and, more importantly, NETs play an important role in the pathogenesis of AAV [11]. NETs can provide a scaffold for platelet and RBC adhesion and concentrate effector proteins involved in thrombosis [12], [13]. In addition, Kambas et al. provided evidence for the release of tissue factor through NETs by neutrophils [14], [15]. Another possible mechanism is associated with neutrophil-derived microparticles, whose level was elevated in active AAV [16]. Kambas et al. found expression of tissue factor in neutrophil-derived microparticles [15]. Tissue factor can induce thrombin generation and thus promote hypercoagulation. Regarding the prothrombin time or activated partial thromboplastin time, which are routine parameters for assessing coagulation, there was no significant difference between AAV patients in active and remission stage in the current study. Therefore, a more accurate index is needed for evaluating the blood coagulation status in AAV patients. For example, Hilhorst et al. used the endogenous thrombin potential (ETP), a sensitive indicator of overall plasma coagulability, to demonstrate hypercoagulability in AAV patients [17], [18]. However, since the current study was a retrospective one, such parameter was not routinely measured.

It was found in our study that patients with elevated D-dimer levels had significantly higher levels of initial serum creatinine, ESR, CRP, BVAS, white blood cell count and platelet count, and significantly lower level of hemoglobulin in the peripheral blood cell count test, compared with patients with normal range of D-dimer. Furthermore, correlation analysis showed that in active phase of AAV, the level of D-dimer correlated with ESR and CRP. These results suggested that the high activity of coagulation and fibrinolysis was associated with active diseases of AAV. The underlying mechanism is not fully clearly yet. There are extensive crosstalks between immune system and the coagulation system [19]. Inflammation can shift the balance promoting a prothrombotic state [20]. The production of the proinflammatory cytokines, such as TNFα, interleukin-1 or C5a, may trigger thrombotic processes by increased expression of tissue factor on endothelial cell and/or neutrophils, which initiates the extrinsic coagulation pathway [21]–[23]. Furthermore, there was a close correlation between disease activity in patients with GPA or MPA and markers of endothelial cell damage [24], and apoptotic endothelial cells have been shown to become procoagulant and proadhesive for platelets [25].

In the current study, the prevalence of VTEs among patients with AAV in active stage was 4.05%, and no patients with AAV in remission developed VTEs. This was in line with some previous studies [4], [5]. However, as a retrospective study, not every patient routinely received imageological examinations to screen thrombosis, we could not accurately assess the real prevalence of VTEs in AAV patients. Furthermore, microthrombus may not be detected in imageological examinations.

In conclusion, this study found that the patients with AAV in active stage were in hypercoagulable state, and circulating level of D-dimer was associated with the disease activity of AAV. The underlying mechanism for this high activity of coagulation system may lie in systemic inflammatory condition.

## Key Messages

Patients with AAV in active stage are in hypercoagulable state. Circulating levels of D-dimer are associated with disease activity of AAV.
